# Owner's Perception of Seizure Detection Devices in Idiopathic Epileptic Dogs

**DOI:** 10.3389/fvets.2021.792647

**Published:** 2021-12-09

**Authors:** Jos Bongers, Rodrigo Gutierrez-Quintana, Catherine Elizabeth Stalin

**Affiliations:** Neurology and Neurosurgery Service, The School of Veterinary Medicine, College of Medicine, Veterinary Medicine and Life Sciences, University of Glasgow, Glasgow, United Kingdom

**Keywords:** seizure detection, sensor, monitoring, wearable, survey, diary, epilepsy, canine

## Abstract

Accurate knowledge of seizure frequency is key to optimising treatment. New methods for detecting epileptic seizures are currently investigated in humans, which rely on changes in biomarkers, also called seizure detection devices. Critical to device development, is understanding user needs and requirements. No information on this subject has been published in veterinary medicine. Many dog health collars are currently on the market, but none has proved to be a promising seizure detector. An online survey was created and consisted of 27 open, closed, and scaled questions divided over two parts: part one focused on general questions related to signalment and seizure semiology, the second part focused specifically on the use of seizure detection devices. Two hundred and thirty-one participants caring for a dog with idiopathic epilepsy, were included in the study. Open questions were coded using descriptive coding by two of the authors independently. Data was analysed using descriptive statistics and binary logistic regression. Our results showed that the unpredictability of seizures plays a major part in the management of canine epilepsy and dog owners have a strong desire to know when a seizure occurs. Nearly all dog owners made changes in their daily life, mainly focusing on intensifying supervision. Owners believed seizure detection devices would improve their dog's seizure management, including a better accuracy of seizure frequency and the ability to administer emergency drugs more readily. Owners that were already keeping track of their dog's seizures were 4.2 times more likely to show confidence in using seizure detection devices to manage their pet's seizures, highlighting the need for better monitoring systems. Our results show that there is a receptive market for wearable technology as a new management strategy in canine epilepsy and this topic should be further explored.

## Introduction

Epilepsy is a common neurological condition in dogs, and management of this chronic disorder requires substantial commitment on the part of the pet owners (1, 2). Seizures can be unpredictable and appear uncontrollable. Many owners therefore express stress and anxiety about their pet having seizures, especially when they are not directly monitoring their pet (3–5). Despite strong dedication, seizure counts based on seizure diaries are often inaccurate and underestimated (6, 7).

New methods for detecting epileptic seizures are currently investigated in humans, which rely on changes in biomarkers during the pre-ictal and ictal phase that are recorded using electronic, wireless and mobile technology, also called seizure detection devices (8–10). Promising results have been reported using accelerometer (for movement detection), heart rate variability, electrodermal activity (skin conduction), and surface electromyography (11–13). In humans combining results of multiple biomarkers via a wrist-worn device has increased overall sensitivity and reduced the false alarm rate (14). A high false alarm rate can lead to alarm fatigue, which occurs when only a small proportion of alarms is relevant and the caregiver subsequently stops responding to alarms as these tend to be “nothing” in most occasions (15). These new devices should lead to a more accurate detection of seizures and in turn are therefore expected to improve monitoring of treatment efficacy.

Sporadic information is available on biomarkers or autonomic changes during the ictal phase in veterinary medicine and there is only one study looking at wearable sensors for seizure detection, which investigates accelerometry using a collar-mounted device (16). There is however a growing interest in wireless and wearable activity monitors for evaluation of general dog health and behaviour. Only a few of these devices have been validated in peer-reviewed publications (17), but they could form footing for a wireless, electronic gadget detecting seizures, using a similar approach as in human medicine.

A critical step in the design of a seizure detection device, is to consider user needs and requirements. No information has been published in veterinary medicine, but several surveys exist on this subject in people. These studies gave insight in perspectives of patients with epilepsy and their caregivers regarding the features and properties of such a device (18–20).

We have performed a survey for owners of epileptic dogs, which consisted of two parts. The first part aimed to investigate the impact of canine epilepsy on daily life, the factors that play a major role in this and the current method for seizure monitoring. The second part included the main purpose of the study and investigated the viewpoints regarding user needs and requirements on seizure detection devices for detecting seizures in dogs.

## Materials and Methods

### Survey Design

A survey was created using an online survey tool [Jisc online tool (21)] (Supplementary Material). It consisted of 27 questions divided over two parts: part one focussed on general questions relating to signalment, diagnosis, seizure onset, seizure description, and seizure management; and the second part focussed specifically on seizure frequency and the opinion of owners on a seizure detection device. The first and second part were divided by a brief explanation of the concept of seizure detection devices to detect seizures and how they are used in people. The survey included open (*n* = 8), closed (*n* = 11) and scale questions (*n* = 13, Likert-type scale, 1–5). For analyses purpose, the scaled questions were either described as individual scales (0 = strongly disagree, 1 = disagree, 2 = slightly disagree, 3 = slightly agree, 4 = agree, 5 = strongly agree) or summarised as “agree” or “disagree.” The answers to the open questions were categorised. Providing an answer was mandatory to continue to the next question. Multiple choice questions were accompanied with the options “Other, please specify” or “I don't know.” The survey was anonymous, and all participants answered voluntarily.

### Recruitment of Responders

The survey was presented online via the online survey software and tool Jisc (http://www.jisc.ac.uk/). The online link to the survey was made available on the research site of the Kennel Club (https://www.thekennelclub.org.uk/) and on several Facebook pages focussing on dogs with epilepsy. Owners of dogs with seizures at the authors' institutions were invited by email which included the link to the website. The survey was available, and data was collected from April 2020 until November 2020. The principal investigators had access to all data for further statistical analysis. Ethical approval was granted by the Ethics Committee of the College of Medical, Veterinary & Life Sciences of the University of Glasgow (Ethical Approval No: 200190049).

### Inclusion Criteria

Responders were included if (1) they gave or had previously given care for a dog diagnosed with idiopathic epilepsy (IE) and (2) if they had completed the survey. Responders were excluded when the aetiology for the seizures was other than idiopathic epilepsy. Data was also excluded if any of the answers did not appear to be truthful which was found in four cases. One response included “keyboard mashing” (“Plohskhabs” was filled in under breed), one response appeared to be randomly filled in (strongly disagreed with all statements and “I don't know” was answered in all open questions) and two responders were removed as a precaution due to questionable authenticity as both answered their dogs had more than 100 seizures per month. The answers were assessed on an individual basis by one of the authors (JB) using the information available from the survey.

### Data Coding

Open questions were coded using descriptive coding to facilitate analyses. This also accounted for the open answer option of the multiple-choice questions. These were either allocated to the predetermined answers or new codes were created. New codes were created independently by two of the authors (JB and CS). These codes were compared and the coding per answer was accepted if there was an agreement in coding between the authors, or after discussion between the authors in cases that differed. Based on the open answers regarding seizure frequency, the answers were allocated to the categories' “0–1 per month,” “2–4 per month,” and “more than 4 per month.”

### Word Cloud Analysis

A visual representation of word frequency of question 21 (“What factors would make it easier to leave your dog at home alone?”) was performed to emphasise the focus of the written material.

### Statistical Analysis

All data was downloaded from the survey software and exported to Microsoft Excel 2013. Statistical analysis was performed using Jamovi [Jamovi version 1.6.15 solid, The Jamovi Project (2021), Sidney, Australia] (22). Descriptive statistics were derived for determining frequencies. Binary logistic regression was performed to test whether the stated level of confidence of managing epilepsy using s for seizure detection (coded as high or low), was associated with the following dependent variables; (1) frequency of seizures (coded as high vs. low), (2) whether responders were monitoring their pet's seizures (coded as yes vs. no), (3) comfortably recognising a seizure (coded as yes vs. no), (4) hours unsupervised during the daytime (codes as unattended <4 h = supervised vs. unattended >4 h = unsupervised), and (5) the frequency of unnoticed seizures (coded as > once a month = high and < once a month = low). Statistical significance was taken as *p* < 0.05 and odds ratios with 95% confidence intervals were calculated for each association. Collinearity of variables was performed to assess correlations between the variables and total model fit assessed by AIC (FIX abbreviation) and McFaddens *R*^2^.

## Results

### Descriptive Data

Two hundred and fifty-eight owners participated in the study. Twenty-three responders were excluded for diagnosis other than idiopathic epilepsy. Another four responses were excluded for untrustworthy data as described above, leading to 231 participants included in the study. Fifty-eight percent of dogs was diagnosed according to IVETF Tier-I and 37% according to IVETF Tier-II. The remaining 5% had no tests performed and 10/11 dogs were between 1 and 6 years of age. The age of seizure onset of 1 dog was unknown according to its owner and this dog was 8 years of age during the time of the study. Sixty-three breeds were included, and the most common breeds were Border collies (*n* = 47), Labrador retriever (*n* = 14), Hungarian vizsla (*n* = 10), Golden retriever (*n* = 8), Greyhound (*n* = 6) and French Bulldog (*n* = 6). The study included 69 females and 128 males; in 34 dogs, information on the gender was not provided by the participant. The mean age was 5.7 years (median 5.0 years, range 0.6–16.0 years).

### Seizure Phenotype

Seizure type was divided into generalised tonic-clonic seizures, focal seizures, or absence seizures, and multiple answers were possible. Most dogs had generalised tonic-clonic seizures (94%) (Table 1). Eight percent displayed generalised and absence seizures, 18% displayed generalised and focal seizures and 12% had all three types of seizures. Most dogs included in this study had a relatively low seizure frequency with 47% displaying only 0–1 seizure per month. But a high number (58%) of the dogs did require hospitalisation for treatment of their seizures at some stage. Eighteen percent had more than 4 seizures per month. Although standard emergency therapy is usually prescribed for use at home following the diagnosis of IE, it was remarkable that almost half (43%) of the caregivers did not administer any additional medication at the time of a seizure. Of the 57% that did administer emergency treatment, 43% used rectal diazepam as emergency medication.

**Table 1:** Survey results—part I.

**Question**	**Result (% of 231 participants)**
**Seizure type**	
Generalised tonic-clonic	218 (94%)
Focal	81 (35%)
Absence	47 (20%)
Hospitalisation for seizure control yes/no	97 (42%)/134 (58%)
**Seizure frequency per month**	
0–1	110 (47%)
1–0	6 (3%)
2–4	73 (32%)
>4	42 (18%)
**Anticonvulsant medication**	
Phenobarbital	145 (63%)
Bromide	57 (25%)
Levetiracetam	79 (34%)
Imepitoin	21 (9%)
Other	36 (16%)
**Additional medication at time of seizure**	
Rectal diazepam	97 (42%)
Levetiracetam	62 (27%)
None	99 (43%)
Monitoring of seizure frequency yes/no	208 (90%)/23 (10%)
**Monitor system for seizure frequency**	
Seizure diary–written or unspecified	85 (43%)
Seizure diary–electronic	44 (22%)
Seizure diary–RVC Pet Epilepsy App	34 (17%)
Unknown	36 (18%)
**How often updating seizure monitoring system**	
On a daily basis	73 (35%)
On a monthly basis	73 (35%)
On a weekly basis	44 (21%)
On a yearly basis	18 (9%)
**Frequency visit to veterinary professional**	
On a regular basis e.g., every 6 months	126 (55%)
Based on changes in seizures	106 (46%)
Only when the pet has a seizure	21 (9%)
Never	7 (3%)
**Changes incorporated following the diagnosis IE**	
More at home/ Increased supervision	175 (76%)
Placed video camera	71 (31%)
Sleeping in the same room	146 (63%)
Refurnished the house to reduce hazards	59 (26%)
Other	43 (19%)
**Hours pet unsupervised daytime (8 a.m.−8 p.m.)**	
0–4	181 (78%)
4–12	50 (22%)
**Hours pet unsupervised night-time**	
0–4	169 (73%)
4–12	62 (27%)
**Seizures suspected to be missed by owner on average**	
None	137 (60%)
Once a week	3 (1%)
Once a month	20 (9%)
Once every 6–12 months	66 (29%)
Unknown	5 (2%)
**Change in actions if alerted that pet has a seizure**	
Administer emergency medication	37 (16%)
Be calmer and prepared	46 (20%)
Be more worried	4 (2%)
Ensure my dog is safe	49 (21%)
Go immediately to my dog	71 (31%)
No changes	24 (10%)

### Impact of Seizures on Daily Life

The participants were asked via a MCQ which changes they had incorporated in their daily life following the diagnosis of IE. Most responders had made changes to monitor their pet's daily activity more closely. It seemed to be important for people to be aware when a seizure had taken place, as 76% of participants increased the amount of supervision of their dogs and 63% slept in the same room. Up to 31% of people installed a video camera following the diagnosis of IE, illustrating the urge to know if and when a seizure occurs. Reducing hazards in the house was implemented by 26% of the responders. Participants were also given the option to either specify their choices or to list any other changes they had made, but these changes in daily life were far less frequent and are therefore not reported in this section. The free section exemplified the intense commitment owners had toward their pet. One responder for example removed all scented products to avoid any seizure triggers, one responder moved to a bungalow to provide a safer environment and one responder stated that their whole life revolved around giving the medication exactly 12 h apart. There was also one participant that answered that she and her husband would never leave their dog alone unless really necessary. In addition, the owner's relative would be asked to attend in certain situations (such as when her husband leaves the house) given the violent seizures the dog can have.

The need for close monitoring is also seen in the amount of time the dogs are supervised per day as around three quarters (78%) of the dogs were unsupervised <4 h a day.

**Figure 1:**
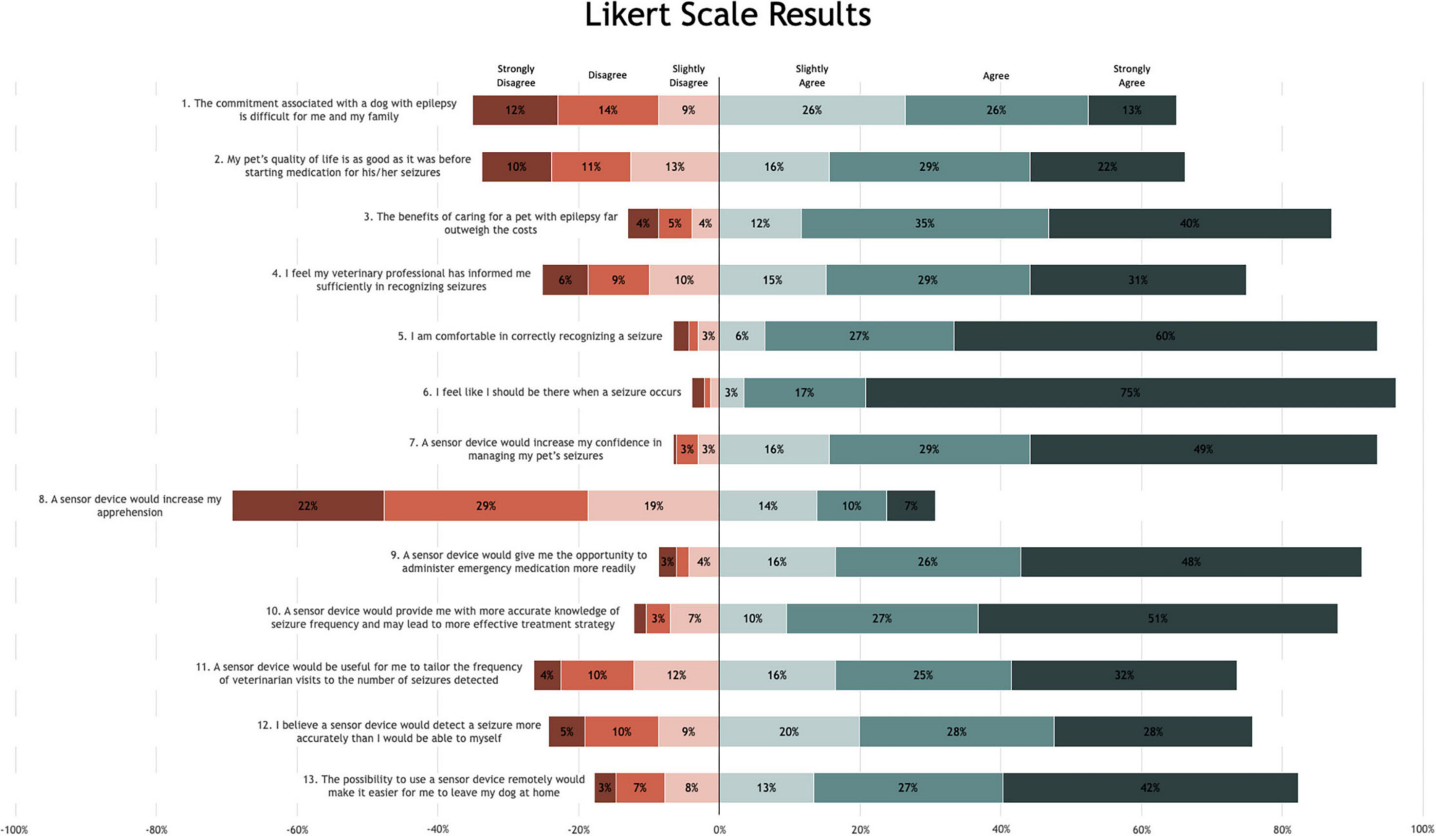
Diverging stacked bar chart—Likert scale results questions 1–13.

Six statements (Likert scale questions) were given during the first part of the study, mostly focussing on seizure recognition and the impact of seizures on daily life (Figure 1, statement 1–6). A striking 95% of dog owners responded that they feel that they should be there when a seizure occurs. Seventy-five percent even strongly agreed with this statement. Considering the above, it is not unexpected that 65% of the participants agreed with the statement “The commitment associated with a dog with epilepsy is difficult for me and my family.” Participants were also asked via a MCQ which change in actions they would undertake if they were alerted that their dog had a seizure. Thirty-one percent would immediately go to their dog and 21% would ensure their dog's safety. Twenty percent answered that they would feel calmer and prepared, compared to a mere 2% that would be more worried. Administering emergency medication as a first change in action was only chosen by 16%. Based on these results, people seemed to feel that it was more important to be with their dog and keep them safe, rather than focussing on stopping the seizures. Despite considerable changes to owner lifestyle 87% felt the benefits of caring for a pet with epilepsy far outweigh the costs.

### Seizure Recognition and Seizure Monitoring System

Correctly recognising a seizure does not seem to be a major problem for owners based on the results of this survey. Around three-quarters of the dog owners felt that their veterinary surgeon had informed them sufficiently on recognising seizures and 93% of the responders were comfortable recognising a seizure with 60% strongly agreeing to this statement. In addition, most dog owners (60%) suspected to never miss a seizure. Seizure recording or tracking was an important part of seizure management amongst the responders and 208 (90%) participants recorded their dog's seizures in some way. Of these 208 responders, 82% used a diary, either written or electronic. The app of the Royal Veterinary College (RVC) was popular amongst the responders and was used in 17% of all diary types. Although only 18% of dogs had more than 4 seizures per month, 35% of dog owners updated their seizure monitor system on a daily basis.

### Seizure Management

**Figure 2:**
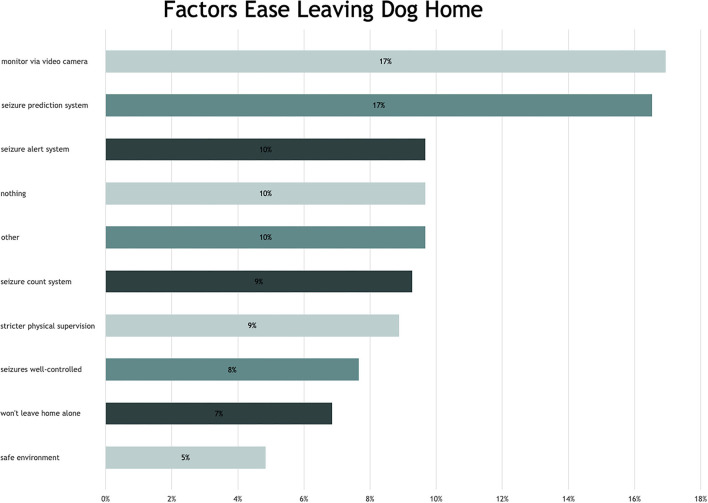
Bar chart—factors that would ease leaving the dog home alone.

For the response to “What factors would make it easier to leave your dog at home alone?” ten codes were defined as an interpretation of owner response (Figure 2). Most dog owners wanted to know if their dog had a seizure whilst being away. There were subtle differences in the reasons for this. Some owners wanted to know if and when a seizure occurred so they could prepare for it (coded: seizure prediction system), other owners wanted to know if a seizure had occurred so they could accurately update their diary (coded: seizure count system) and some owners wanted to be alerted when their dog had a seizure so they could go home to be with their dog (coded: seizure alert system).

**Figure 3:**
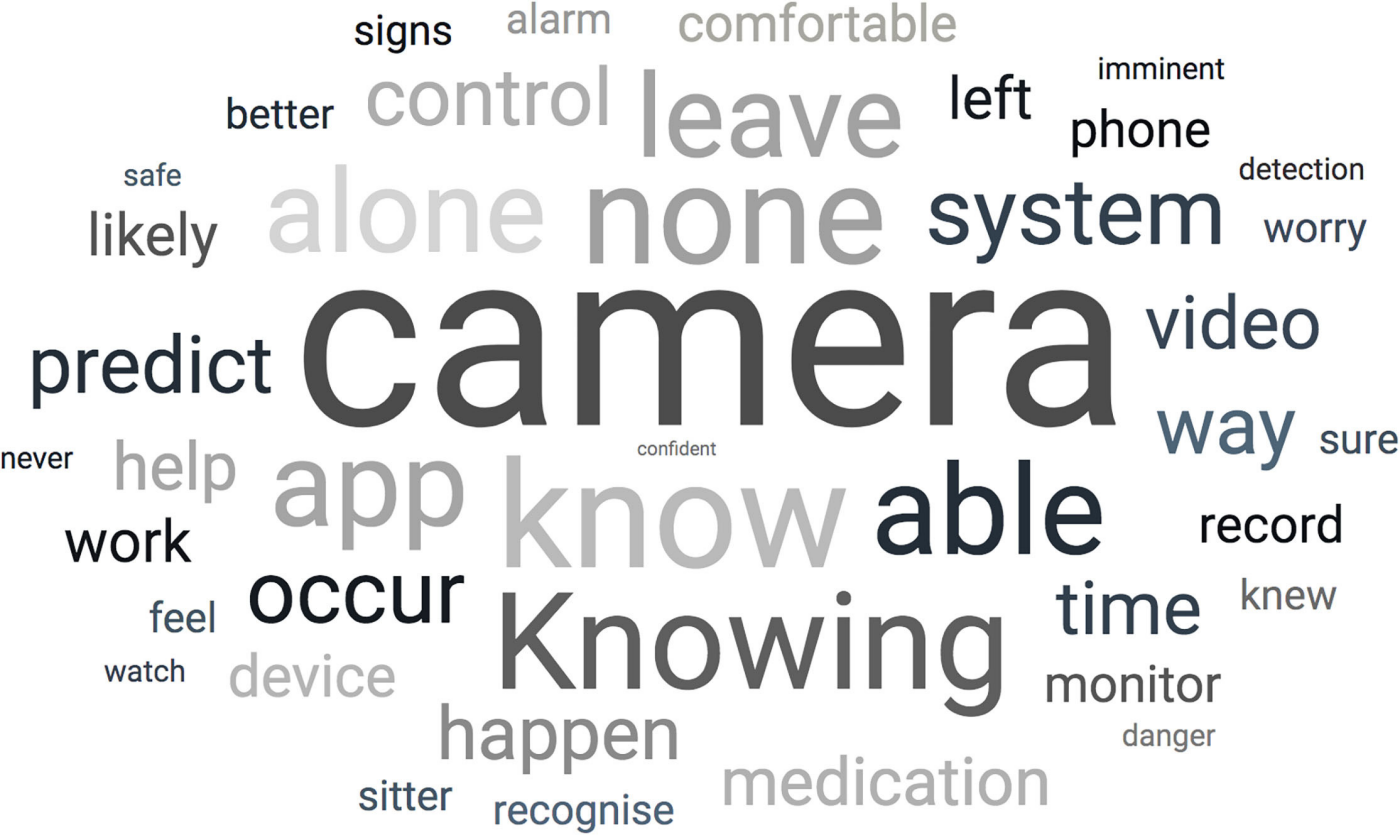
Word cloud—open answers to the question “What factors would make it easier to leave your dog at home alone?”.

Eleven percent of the responders felt nothing would make it easier whereas 17% felt that having a video recorder and a further 17% felt that being able to predict a seizure would make it easier to leave their dog at home. Frequently used words to promote leaving the dog unassisted included “know(ing)” and “camera” and the key component for owners was to know what to expect (Figure 3).

### Seizure Detection Devices

There was an overall positive response toward the idea of using seizure detection devices (seizure sensors) to detect seizures as management strategy. Most people felt seizure detection devices could help in seizure management. A seizure detection device using sensors would increase the confidence of 93% of the responders in managing their dog's seizures with 49% strongly agreeing to this statement. In addition, 88% believed a seizure detection device would provide more accurate knowledge of seizure frequency and 82% felt that using a seizure detection device remotely would make it easier for them to leave their dog alone at home. Most of the participants also felt a seizure detection device would enable them to administer emergency medication more readily. Although over 90% of the participants felt confident in recognising a seizure, most dog owners (76%) believed a seizure detection device would detect a seizure more accurately (Figure 1). Most owners (89%) preferred to use a seizure detection device to help count their dog's seizures, over 26% of owners opted visual counting via watching video footage.

Participants were also asked via MCQs (multiple answers possible) where the detection device should be worn and how the data should be recorded. Seventy-one percent preferred the sensor to be worn as a collar around the neck, 23% as an intracranial implant via a mini operation, 17% as part of a harness and 10% around the paw. Around half (48%) of the dog owners preferred the data to be uploaded from the sensor and 47% preferred to read the data directly from the sensor. Only 5% opted to directly send the data to their veterinary professional, illustrating that owners prefer to receive this information directly without involvement of their veterinarian first. The concept of “false alarm” was also explained to the participants and they were questioned how many false-positive results would be acceptable. People had a high tolerance for false alarms and not missing a single seizure seemed paramount. An impressive 52% will tolerate any false alarm rate (FAR), if no seizure was missed. One third (30%) had a different opinion and would accept a FAR of only <1 per week. Our last question gave us valuable new insights into owners' views on the features and properties of seizure detection systems. They were asked via an open question their main concerns with regards to different types of sensors. The answers were coded, and these codes could be divided into three different categories, including an internal device, an external device, or all devices (Table 2). There were striking similarities amongst the answers and the following response summarises it well:

“*An extra cranial device is likely to be damaged through normal dog activity e.g., swimming, running through undergrowth. She is likely to chew anything around her paw and she does not wear a collar and harness all the time in the house. I would prefer an intra cranial device because it is less likely to be damaged and it would be a permanent fixture. The drawbacks for the intracranial implant would be around the risk of the operation and cost.”*

**Table 2:** Survey results—part II.

**Question**	**Result (% of 231 participants)**
**Preferred monitor system (SCQ)**	
Seizure detector via sensors	205 (89%)
Video system (e.g., baby camera)	59 (26%)
Intensify contact with local veterinary professional	15 (6%)
**Acceptable false positive rate**	
1 per day	3 (1%)
1 per week	28 (12%)
2–4 per week	12 (5%)
< 1 per week	69 (30%)
As many as it takes, as long no seizure is missed	119 (52%)
**Seizure data recording**	
All data directly emailed to my veterinarian	12 (5%)
Automatic diary uploaded from the sensor	111 (48%)
Read directly from the sensor	108 (47%)
**Where should the sensor be worn**	
Collar around the neck	163 (71%)
Collar around the paw	23 (10%)
As part as a harness	40 (17%)
As an intracranial implant (within skull)	52 (23%)
Other	8 (3%)
**Advantages and disadvantages of different sensor types**	
All sensors—may aggravate epilepsy	53 (23%)
External device—potential hazards	
External device—less accurate	25 (11%)
External device—easier to use/wear/replace	48 (21%)
External device—stress and discomfort	12 (5%)
Internal device—invasiveness, stress and complications of operation	126 (55%)
Internal device—costs	13 (6%)
Other	18 (8%)

### Predicting the Level of Confidence Using a Sensor Using Logistic Regression

Using logistic regression, we did not identify any significant association between seizure frequency, level of comfort in recognising a seizure, the number of hours unsupervised during the daytime, the frequency of unnoticed seizures, and the confidence in using a seizure detection device for managing their pet's seizures. We did however identify a significant association between whether the responders were monitoring their pet's seizures, and the confidence in using a seizure detection device for managing their pet's seizures. Monitoring the seizures was associated with increased odds (4.2 times) of having a high confidence seizure management by using a seizure detection device (Table 3).

**Table 3:** Associations between survey responses and the stated level of confidence managing seizures using a seizure detection device determined by multivariate binomial logistic regression.

						**95% confidence interval**	
**Predictor**	**Estimate**	**SE**	**Z**	***p***	**OR**	**Lower**	**Upper**
Keeps track of seizures (yes vs. no)	1.4	0.6	2.2	0.025	4.2	1.2	14.4
Seizure frequency (high vs. low)	−0.4	0.6	−0.6	0.531	0.7	0.2	2.3
Unsupervised during day (vs. supervised)	−0.8	0.6	−1.4	0.155	0.4	0.1	1.4
Comfortable in recognising a seizure (yes vs. no)	16.2	1578.6	0.0	0.992	1.14e+7	0.0	Inf
Missed seizures (many vs. few)	0.3	0.8	0.3	0.745	1.3	0.3	6.9

*Inf, infinite; Odds ratios represent the odds of a predictor being associated with a high level of confidence in using a seizure detection device*.

## Discussion

Our survey investigated the viewpoints of dog owners toward the use of seizure detection devices to assist in the management of canine epilepsy. Our results show that dog owners have a high confidence in using seizure detection devices to improve seizure management. People were highly invested in finding ways to better manage their dog's epilepsy and knowing when a seizure occurred was paramount. Most owners kept track of their dog's seizures daily or monthly and were anxious to leave their dog alone given the unpredictability of seizures. Seizure management mainly centred on knowing when a seizure occurred and being there for their pet to ensure their safety, rather than focussing on how to improve long-term medication or administrating emergency treatment. Only half of the owners for example, routinely administrated emergency medication when a seizure occurred. Owners felt they would have better control over their dog's seizures if they knew when their dog had a seizure, for example by monitoring them via a video camera or by a seizure detection device that would alert them if a seizure was imminent.

Owners preferred to use a sensor device to predict seizures over a device that would count seizures or would alert them if a seizure was happening. However, a relatively high percentage (26%) preferred to use a video camera over a sensor device as a monitor system. Using a video camera may give a better sense of control as you are directly visualising your pet as opposed to trusting a device but it is more laborious as this requires analysis of the full footage and manual counting of any seizure activity seen within the reach of the camera. Seizure activity outside this reach would be missed. Video detection systems have been used in people but they rely on automatic interpretation of video data (23, 24). These are largely grouped into marker-based or marker-free systems. Marker-based systems use cameras that track easily detectible objects of various shapes and sizes and are placed on motion relevant positions such as joints or extremities. Marker-free systems rely on the content of image sequences taken by one or more video cameras and is analysed automatically (25). Both methods however recognise mainly seizures with large movements. In addition, marker-based sensors can be uncomfortable or dislocate over time and marker-free systems are limited to the area covered by the video camera and the patient must be clearly visible without any objects in the way (26).

Previous studies have investigated the impacts of owning a dog with idiopathic epilepsy on owner quality of life (2–4, 27, 28). Qualitative and quantitative studies have shown that the majority of owners caring for a dog with epilepsy, have made lifestyle changes in order to care for their dog and there is a fear of leaving their dog unsupervised (28). This is similar to our results which show that the vast majority of owners (98%) have incorporated changes in their daily life. Increased supervision following the diagnosis of IE, plays a major role as 76% of the participants slept in the same room and 31% placed a video camera. In addition, 89% of the participants kept track of their dog's seizures. These findings suggest supervision and seizure monitoring have a prominent place in caring for an epileptic dog. A seizure detection device which enables owners to monitor their pet's seizure activity remotely may improve the impact owning a pet with epilepsy has on the owner's life. Certainly, this study shows that 88% of owners would be more willing to leave their pets unattended if a sensor was in place.

Statistical analysis revealed only one correlation, which showed owners that monitored their dog's seizures were 4.2 times more likely to have a high confidence in managing their dog's epilepsy using a seizure detection device. This is not surprising as owners that are already documenting their dog's seizure activity are likely to be open for other methods that make it easier or are even more accurate in seizure reporting. We did however expect to find more correlations, such as a positive correlation between a high level of confidence in managing the seizures using sensors and owners that were not very comfortable in recognising a seizure or pet's that were unsupervised throughout the day. A reason for this can be explained by the few responses in the low confidence group; only 7% was not of opinion that a seizure detection device would increase their confidence in managing their dog's epilepsy. This could for example be due to the study being underpowered or to sample bias toward “high confident” owners. Given our sample size, we suspect the latter is more likely.

Most of our responders felt the sensor should ideally be worn externally to avoid the stress and complications of a surgery. We suspected a collar around the neck would be the preferred location as many dogs would wear a collar regardless, however we did not anticipate the high number of participants that commented on the potential hazards an external device could bring. This should be taken into consideration during the design of the device. The false alarm rate (FAR) seems to be of less importance for dog owners when compared to caregivers for people as our results show that 52% of the participants will accept any FAR if there are no false negatives (illustrating the urge to be informed when their dog has a seizure). This number is very likely to be lower after using the device as our participants had not experienced “*alarm fatigue*.”

The results from our open questions show that many dog owners would like to be informed in some way on their dog's seizure activity. They would like to be alerted when a seizure occurs, they would like to know that a seizure has occurred (this may be at the time of the seizure or afterwards) and they want to be able to predict that a seizure is going to occur. Some answers explicitly made the distinction between seizure prediction and seizure monitoring or alerting, but in other occasions this distinction could not be confidently made. Although our study focusses on the implication of a device that detects or registers seizures as explained to the participants in the introduction of the second half of the survey, this may have been interchangeably used by some owners with a seizure prediction device. A seizure prediction device may provide owners the opportunity to give preventive medication for a potential seizure. Little is known about veterinary seizure prediction methods, and they mostly rely on behavioural changes before a seizure. The results of a recent survey showed that over half (59.6%) of owners surveyed, believed that they were able to predict forthcoming seizure activity (29). However, the accuracy could not be verified, and the sensitivity and specificity remain unknown. There are also studies that have investigated the predictability of seizures using intracranial electroencephalographic (iEEG) measurements (30–32). Although these results are promising, it is unlikely this will be implemented in the near future as it is invasive, costly and requires specialised expertise in placing the device and reading the results. Moreover, many dog owners in our study declined a surgery due to the invasiveness. A non-invasive system to predict seizures could rely on physiological changes before a seizure occurs. So far there are no studies that have investigated physiological changes during the preictal or ictal period, however, there has been a study published looking at changes during the interictal period. This study found a significant difference in heart rate variability between dogs with presumed IE and healthy dogs as dogs with presumed IE had an increased activity of the parasympathetic component of the autonomic nervous system (33). This can be considered when determining thresholds for seizure detection devices.

Our study has several other limitations. One bias regards the population of owners surveyed (sample bias). Dog owners with stronger commitment toward their epileptic dog may have been more likely to participate and in addition, the most common dog breed was the Border collie. Management of IE in this breed has been described as often challenging, requiring a stronger owner commitment (34). This may have influenced our results, although only 18% of the dogs in our study had more than 4 seizures per month. Participants were also not obligated to provide proof of diagnosis and therefore the diagnosis was based on owners' interpretation. Lastly, to avoid gaps in the data every question required an answer before moving to the next question which may have resulted in participants choosing the most appropriate answer however it may not have reflected their true opinion.

## Conclusion

The unpredictability of seizures plays a major part in the management of canine epilepsy and dog owners have a strong desire to know when a seizure occurs. Our study on dog owners' perception on seizure detection devices showed that owners believed seizure detection devices would improve their dog's seizure management, including a better accuracy of monitoring seizure frequency and the ability to administer emergency drugs more readily. A seizure detection device was the preferred method of seizure monitoring system and a wearable device was preferred over an implant. Half of the responders accepted any FAR provided no seizure was missed, though the FAR is one of the major limitations in people after having used seizure detection devices in practise. Another seizure management strategy frequently mentioned was seizure monitoring via video cameras. This however would require a manual seizure count and seizures can only be captured if the patient is visible and properly placed within the area covered by the video camera. These findings illustrate the need for new seizure management strategies in veterinary medicine in which wearable technology may have a prominent place in improving seizure monitoring and reducing owner anxiety. This in turn aims to improve canine epilepsy management by boosting owner confidence and therefore overall increasing the quality of life of the dog and its carer. Our study also provides suggestions which should be taken into consideration when developing such a device. An important factor is that the device should be reliable. The study can be used as a starting point for future studies that focus on non-EEG parameters in veterinary research.

## Data Availability Statement

The raw data supporting the conclusions of this article will be made available by the authors, without undue reservation.

## Ethics Statement

The studies involving human participants were reviewed and approved by Ethics Committee of the College of Medical, Veterinary & Life Sciences of the University of Glasgow. The patients/participants provided their written informed consent to participate in this study. Written informed consent was obtained from the owners for the participation of their animals in this study.

## Author Contributions

JB conceived the original idea and recruited responders. JB and CS designed the survey. JB wrote the manuscript with support from CS and RG-Q. CS supervised the project. All authors contributed to the article and approved the submitted version.

## Conflict of Interest

The authors declare that the research was conducted in the absence of any commercial or financial relationships that could be construed as a potential conflict of interest.

## Publisher's Note

All claims expressed in this article are solely those of the authors and do not necessarily represent those of their affiliated organizations, or those of the publisher, the editors and the reviewers. Any product that may be evaluated in this article, or claim that may be made by its manufacturer, is not guaranteed or endorsed by the publisher.
