# Supplementary material for: Owner's Perception of Seizure Detection Devices in Idiopathic Epileptic Dogs
Source: Front Vet Sci. 2021 Dec 9;8:792647. doi: 10.3389/fvets.2021.792647 (PMC8711717; doi:10.3389/fvets.2021.792647)
Supplement: Supplementary file 1 [file Data_Sheet_1.PDF]

SURVEY for STUDY ON NOVEL SEIZURE SENSOR DEVICES

Page 1: PARTICIPATION INFORMATION

Survey to investigate the need for a sensor device that can predict seizures in dogs in the home setting.

You are being invited to take part in a research study. Before you decide, it is important for you to understand why the research is being done and what it will involve. Please take the time to read the following information carefully and discuss it with others if you wish. Ask us if there is anything that is not clear or if you would like more information.

What is the purpose of the study?

The aim of this survey is to better map the impact of the unpredictable occurrence of seizures on the caregivers and investigate the need for and confidence in seizure detection devices. A seizure sensor device is a sensor using autonomic parameters (e.g. heart rate, accelerometry, blood pressure, and skin temperature) to predict seizures and to alert pet owners in order to promote early intervention. The survey will be open until April 2021.

Why have I been invited to participate?

You have been invited to take part in this study because your pet has previously been diagnosed with epilepsy and displays or displayed generalized tonic-clonic seizures (GTCS).

Do I have to take part?

Taking part is entirely voluntary. If you decide to take part, you are still free to withdraw at any time and without giving a reason.

What will happen to me if I take part?

We are aiming to recruit around 225 responders. When we have enough people taking part in this study, we will not include or invite any more. Filling out this questionnaire may take around 15 to 20 minutes. The first part includes general questions and the second part will mainly focus on questions regarding seizure sensors. The survey is anonymous and consists of open, closed, multiple-choice, and scale questions. The survey will be available online (also via a QR-code) and via a paper version available at general veterinary practices in Glasgow and surroundings (+/- 30-mile diameter). The survey will be open from April 2020 through April 2021. Results will be included in a systemic review paper on seizure sensor devices in dogs and this paper will be expected to be submitted for publication at the end of 2021. As the study is anomalous, you will not be personally informed of the results of the study.

What are the possible disadvantages and risks and possible benefits of taking part?

There are no potential disadvantages or harms in taking part in the study. You will also receive no direct benefit from taking part in this study. The information that is collected during this study will give us a better understanding of the need for seizure detection devices in dogs.

Will my taking part in this study be kept confidential?

The information provided by you is completely anomalous and all information which is collected about you, or responses that you provide, during the research will be kept strictly confidential. Any data in paper form will be stored in locked cabinets in rooms with restricted access at the University of Glasgow. All data in electronic format will be stored on secure password-protected computers. No one outside of the research team or appropriate governance staff will be able to find out your name, or any other information which could identify you.

Who is organizing and funding the research?

The study has been funded by the Veterinary School Vet Fund and reviewed by the College of Medical, Veterinary & Life Sciences Ethics Committee.

For further information, please contact j.bongers.1@research.gla.ac.uk.

Thank you very much again for participating in our survey. Your contribution is very

important to us and this data will be used for future studies.

Page 2: INTRODUCTION

Epilepsy is a common problem in dogs, and management of this chronic disorder requires a substantial commitment on the part of the pet owner. Seizures can be unpredictable and appear uncontrollable. Many caregivers express stress and anxiety about their pets with epilepsy having seizures when they are not present. Despite strong dedication, seizure diary reported seizure counts are often inaccurate and underestimated. Therefore, a need exists for the ability to recognize and/or detect a seizure in the home setting. New methods for predicting seizures are currently investigated in humans, such as changes in autonomic function (e.g. heart rate or blood pressure) but also electronic, wireless, and mobile seizure detection devices are increasingly used to monitor vital parameters during seizures. It would be interesting to investigate if this is also applicable to dogs. The aim of this survey is to better map the impact of the unpredictable occurrence of seizures on the caregivers and investigate the need for and confidence in seizure detection devices.

This study is funded and executed by the University of Glasgow.

Filling out this survey may take around 15 to 20 minutes. The first part includes general questions and the second part will mainly focus on questions regarding seizure sensors. Before starting the survey, we advise you to collect your pet's epilepsy dairy (if available) and the medication of your pet. The survey is anonymous and consists of open, closed, multiple-choice and scale questions. The scale questions range from 0 to 5. This is explained by:

- 5: Strongly agree
- 4: Agree
- 3: Slightly Agree
- 2: Slightly disagree
- 1: Disagree
- 0: Strongly Disagree

The last page of the survey is available for you to leave any questions or comments.

Thank you very much again for participating in our survey. Your contribution is very important to us and this data will be used for future studies.

Page 3: CONSENT

1. I have read and agree to participate anonymously in this survey ✱ *Required*

Please select at least 1 answer(s).

- [ ] Yes

Page 4: PART 1

2. Please provide the breed, gender, and age of your pet (actual or estimated). ✱ *Required*

3. How old was your pet when the seizures started? ✱ *Required*

4. Has a veterinarian determined a cause of your pet's seizures? ✱ *Required*

- ☐ Idiopathic (unknown origin)
- ☐ Eating poison
- ☐ Liver disease
- ☐ Low or high blood sugar
- ☐ Kidney disease
- ☐ Head injury
- ☐ Encephalitis
- ☐ Strokes
- ☐ Brain tumor
- ☐ I don't know

5. Has your pet had any of the following tests to determine the cause of seizures? ✱ *Required*

- [ ] Routine blood test
- [ ] Specific liver tests (e.g. pre-and postprandial bile acids or ammonia)
- [ ] Blood titres for infectious diseases (Toxoplasma gondii or Neospora caninum)
- [ ] MRI scanning of the brain
- [ ] None of the above tests have been done
- [ ] Other

5.a. If you selected Other, please specify:

[ ]

6. What type of seizures does your pet have? ✱ *Required*

- [ ] Generalized tonic-clonic (whole body involved, loss of consciousness, limbs may twitch, jerk or paddle and are often rigid)
- [ ] Focal/ partial seizures (usually conscious, not the whole body but only a small area is affected such as twitching and jerking in one side of your pet’s body and/or face, dog’s trunk curving to one side or head turning to the other)
- [ ] Absence seizure (difficult to recognize but usually brief lasting for about a minute, e.g. staring, lip licking, eyes rolling)

7. Has your pet ever required hospitalization because of his/her seizures? ✱ *Required*

- ○ Yes
- ○ No

8. Over the last 6 months, how many seizures did your pet have each month

on average? ✱ *Required*

9. If your pet has less than one seizure per month on average, what is the average time interval (e.g. in weeks or months) between seizures? ✱ *Required*

10. What oral medication is your pet receiving currently for his/her seizures? ✱ *Required*

11. Do you administer additional medication at the time of a seizure? ✱ *Required*

- ☐ No
- ☐ Yes, Stesolid/Rectiole (diazepam) rectally
- ☐ Yes, Keppra (levetiracetam)

12. Do you monitor your pet's seizure frequency (e.g. by means of a diary of a mobile phone app)? (Yes/no) If yes, please specify? ✱ *Required*

12.a. How often do you keep track of your monitoring system? ✱ *Required*

- [ ] Yes, on a yearly base
- [ ] Yes, on a monthly base
- [ ] Yes, on a weekly base
- [ ] Yes, on a daily base
- [ ] I do not keep track of my pet's seizure activity

13. How often do you take your pet to your veterinary professional with regard to his/her seizures? ✱ *Required*

- [ ] On a regular base, e.g. every 6 months
- [ ] Only when my pet has a seizure
- [ ] Based on changes in seizure frequency or seizure appearance
- [ ] Never

14. Have you as a caregiver routinely incorporated any of the following ✱ *Required*

More info

- [ ] Spend more time at home to be able to supervise my pet
- [ ] Sleep in the same room instead of separate rooms
- [ ] Placed a video camera (e.g. baby camera)
- [ ] Refurnished the house to reduce any hazards
- [ ] Other

14.a. If you selected Other, please specify:

15. What kind of support from your veterinary professionals to help you manage your pet’s long-term care do you receive? ✱ *Required*

- ☐ None
- ☐ I only received information at the time of diagnosis
- ☐ I only visit my veterinarian in case I have any concerned about my pet’s seizures
- ☐ I visit my veterinarian on a regular base for my pet’s epilepsy

16. Please answer the following statements according to the Likert scale ✱ *Required*

Please don't select more than 1 answer(s) per row.

Please select at least 6 answer(s).

	0: Strongly Disagree	1: Disagree	2: Slightly disagree	3: Slightly Agree	4: Agree	5: Strongly agree
The commitment associated with a dog with epilepsy is difficult for me and my family	☐	☐	☐	☐	☐	☐
My pet’s quality of life is as good as it was before starting medication for his/her seizures	☐	☐	☐	☐	☐	☐
The benefits of caring for a pet with epilepsy far outweigh the costs	☐	☐	☐	☐	☐	☐

I feel my veterinary professional has informed me sufficiently in recognizing seizures	☐	☐	☐	☐	☐	☐
I am comfortable in correctly recognizing a seizure	☐	☐	☐	☐	☐	☐
I feel like I should be there when a seizure occurs	☐	☐	☐	☐	☐	☐

17. How many hours per day is your pet unsupervised during the daytime (8 am – 10 pm)? ✱ *Required*

- ☐ 0
- ☐ 1-2 hours
- ☐ 2-4 hours
- ☐ 4-8 hours
- ☐ 8-2 hours

18. How many hours per day is your pet unsupervised during the night-time (10 pm – 8 am)? ✱ *Required*

- ☐ 0
- ☐ 1-2 hours
- ☐ 2-4 hours
- ☐ 4-8 hours

- [ ] 8-12 hours

19. How many seizures do you suspect you potentially miss on average (e.g. when your pet is home alone or unsupervised)? ✱ *Required*

- [ ] None
- [ ] Once every 6-12 months
- [ ] Once a month
- [ ] Once a week
- [ ] > Once a week

20. What factors would make it easier to leave your dog at home alone? ✱ *Required*

21. How would your actions change if you were alerted your dog was about to have a seizure? ✱ *Required*

Page 5: PART 2

One of the most disabling aspects of epilepsy is the unpredictability of epileptic seizures. The use of a detecting device (*sensor device*) linked to an electronic diary could be of practical benefit in seizure management. Seizure detection studies have focused on detecting physiological changes that occur before and during a seizure. Many of these studies have been performed in human medicine and we are currently looking into its application in veterinary medicine.

22. Please answer the following statements according to the Likert scale. ✱ *Required*

Please don't select more than 1 answer(s) per row.

Please select at least 7 answer(s).

	0: Strongly Disagree	1: Disagree	2: Slightly disagree	3: Slightly Agree	4: Agree	5: Strongly agree
A sensor device would increase my confidence in managing my pet’s seizures	☐	☐	☐	☐	☐	☐
A sensor device would increase my apprehension	☐	☐	☐	☐	☐	☐
A sensor device would give me the opportunity to administer emergency medication more readily	☐	☐	☐	☐	☐	☐

A sensor device would provide me with more accurate knowledge of seizure frequency and may lead to more effective treatment strategy	☐	☐	☐	☐	☐	☐
A sensor device would be useful for me to tailor the frequency of veterinarian visits to the number of seizures detected	☐	☐	☐	☐	☐	☐
I believe a sensor device would detect a seizure more accurately than I would be able to myself	☐	☐	☐	☐	☐	☐
The possibility to use a sensor device remotely would make it easier for me to leave my dog at home	☐	☐	☐	☐	☐	☐

23. Which monitor system would you prefer? ✱ *Required*

- [ ] Video system (e.g. baby camera)
- [ ] Seizure detector via sensors
- [ ] Intensify contact with my local veterinary professional

24. How many false-positive results ('false alarm') would be acceptable for you? ✱ *Required*

- [ ] As many as it takes, as long I don't miss a seizure
- [ ] < 1 per week
- [ ] 1 per week
- [ ] 2-4 per week
- [ ] 1 per day
- [ ] 2-4 per day

25. How would you prefer the seizures recorded? ✱ *Required*

More info

- [ ] Read directly from the sensor
- [ ] Automatic dairy uploaded from the sensor
- [ ] All data directly emailed to my veterinarian
- [ ] Other

25.a. If you selected Other, please specify:

26. How should the sensor device be worn? ✱ *Required*

➕ More info

- ☐ Collar around paw
- ☐ Collar around the neck
- ☐ As part of a harness
- ☐ As an intracranial implant (within the skull) via a mini operation
- ☐ Other

26.a. If you selected Other, please specify:

26.b. Please elucidate your above answer. Why would you prefer an implant or a wearable sensor device? What can you see as the disadvantages of each? ✱ *Required*

27. Please let us know below if you have any questions or comments on the previous questions. ✱ *Required*

Page 6: THANK YOU FOR TAKING THE TIME TO COMPLETE THIS SURVEY.
